# The Importance of Definitive Diagnosis in Chronic Schistosomiasis, with Reference to *Schistosoma haematobium*

**DOI:** 10.1155/2012/761269

**Published:** 2012-05-30

**Authors:** Clive Shiff

**Affiliations:** Department of Molecular Microbiology and Immunology, Johns Hopkins Bloomberg School of Public Health, 615 North Wolfe Street, Baltimore, MD 21205, USA

## Abstract

Schistosomes are long-lived parasites, hence schistosomiasis is a chronic disease with severe long-term implications. However, definitive diagnosis of active infection has been difficult because demonstration of infection has depended on detecting parasite eggs in urine and/or stool. In the case of *Schistosoma haematobium* which parasitizes the urinogenital system, this method has low sensitivity in adults. Detection of parasite-specific DNA in urine has been demonstrated and this has similar specificity but improved sensitivity. The implications of this new procedure and the impact on diagnosis are discussed.

## 1. Background and Introduction

In Africa, the health impact of schistosomiasis, whether caused by *Schistosoma haematobium* or by *S. mansoni*, is a well-known public health problem, one that is now receiving well-deserved attention [1]. This attention is focused primarily on the most vulnerable part of the community, that part of the population that is heavily debilitated by the disease and will benefit from mass drug administration. For the purpose of control and local elimination of the parasites, a quick sensitive test that may be low in specificity is acceptable but there is need to improve the detection of infection in chronic stages of disease or when parasitaemia is low. The detection of infection among adults with long standing chronic infections is important particularly in the hospital environment when sequelae of the infection are suspected. Bladder damage and even bladder cancer are common problems in endemic areas [2] and a definitive diagnosis which has high sensitivity, specificity, and can be carried out in a diagnostic laboratory with adequate facilities is needed. Definitive diagnosis of schistosomiasis is dependent on the demonstration of parasite eggs in urine or stool, and more recently the detection of circulating antigens [3]; however, these tests have not been assessed in adults. Detection of parasite-specific DNA is an option that is being used with several infections for example, malaria [4], and it presents an opportunity with *Schistosoma haematobium *[5] and perhaps with *S. mansoni* [6]. The importance of this has been shown in a recent study involving *S. haematobium, *it was evaluated using latent class modeling and was shown to detect parasite-specific DNA fragments in adults both when eggs were present in urine and in 10% of cases, where eggs were not present [7].

Gelfand [8] in a very careful clinical analysis of mainly adults infected with bilharzia (schistosomiasis) concluded that “In Rhodesia (now Zimbabwe) bilharziasis, both the urinary and intestinal is to be regarded as having serious consequences,” he was speaking about the infection in adults as well as children. In a more recent review, King and Dangerfield-Cha [9] reiterate this importance although they do not cover the material in clinical detail as does Gelfand (loc cit). More specifically and focusing on *S. haematobium* the role of this species in bladder cancer is well studied [2], not only in Egypt but also in Kenya [10], Ghana [11], and Zimbabwe [12]. Whereas in children this infection causes haematuria and frequently bladder polyposis, these problems ameliorate following treatment [13]; however, the more severe squamous cell carcinoma appears in the 3rd and 4th decades of life [2].

## 2. Is This a Problem of Concern?

The methods we have used to diagnose schistosomiasis decrease in sensitivity in adulthood and the question arises, are the current diagnostic tests sufficiently sensitive to detect infection in all age groups? Community-based surveys done using the presence of schistosome eggs in urine or faeces as positive infection always show a similar population trend. Prevalence rises to a peak during the years 10–15, then declines through the 20s, 30s and 40s to well less than half of the childhood peak. This surely indicates that the parasite causes the greatest health impact among children, but does it? As lesions form around the schistosome eggs, particularly in the bladder, granulomas develop and egg passage to the exterior becomes hindered. There is a strong inflammatory response from the host and over time metaplasia sets in and eventually the chronic inflammation initiates the development of cancer. Ultrasound examinations done in the Ghana study certainly show the extent of bladder damage in adult bladders [11]; yet there were numerous people in ages over 30 years who showed severe damage but no evidence of eggs in the urine. A detailed study of the sensitivity and specificity of various diagnostic tests used in this study included haematuria, antigen detection, egg detection, and antibody detection but the results were equivocal [14] and indicated the need of a more sensitive, yet specific test for improved diagnosis of schistosome infection.

## 3. Parasite-Specific DNA in Urine: A New Test Procedure

Detection of parasite DNA in blood specimens is now an accepted procedure, whether the DNA is intracellular or extracellular, the presence of trace but detectable quantities of specific fragments provides evidence of the organism. Malaria parasites can be detected in haemolysed blood specimens, but an advance occurred when *Plasmodium falciparum*-specific DNA was also demonstrated in saliva and urine [4]. This DNA was free in saliva and in the urine clearly passed through the kidneys prior to excretion and was undamaged. Collecting urine for subsequent PCR examination has logistic issues as the urine must be fixed or frozen rapidly to −20°C for storage and transport. Operating on the hypothesis that schistosome-specific DNA is passed in urine, we proposed that it could be trapped in a convenient paper filter. As such, this would obviate considerable handling problems and it was shown to be the case. A 50 mL specimen of urine was passed through coarse filter paper (Whatman no. 3) GE Healthcare, Bucks, UK. The paper is sturdy and will hold a cone when folded, filtration could be processed in the neck of a disposable vessel, the paper subsequently dried away from aerial and insect contamination and if maintained dry, the DNA would be preserved. The work was tested in Niger and Nigeria, and proved to be effective [7].

## 4. The Role of *Schistosoma haematobium* Specific DNA Fragment: Example of a New Test

Workers in Kenya and Israel had identified a specific fragment of DNA (*Dra*1) that was detectable from snails infected with *S. haematobium* miracidia [15]. The fragment is specific for *S. haematobium* and was shown to be more sensitive than egg detection or haematuria with high specificity, particularly among adults [5] where egg detection versus PCR showed a sensitivity of 59%. This infers that among adults, egg detection is unsatisfactory and supports the comment made above. Analysis of results from a large-scale epidemiological study comparing three measurements, haematuria, presence of parasite eggs, and detectable parasite-specific DNA using latent class modeling was undertaken [7]. This is a statistical technique that models the probability of each combination of test results to give the true infection status (this is the latent class variable—true infection—which is unobservable). This model provides response probabilities for sensitivity (Se) and specificity (Sp) for each of the test procedures and finally indicates statistically which test is the most sensitive (i.e., with fewest false positives) and most specific (with fewest false negatives). It was shown that presence of *Dra*1 in males exceeded haematuria (Se 87.6% and Sp 34.7%) and detection of eggs (Se 70.1% and Sp 100%). In females, presence of *Dra*1 exceeded haematuria (Se 86.7% and Sp 77%) and presence of eggs (Se 70.1% and Sp 100%). Furthermore, *Dra*1 became undetectable 2 weeks after praziquantel treatment. This suggests that detection of *Dra*1 is a definitive test for the presence of *S. haematobium *infection.

## 5. Significance of the New Diagnostic Test

**Figure 1:**
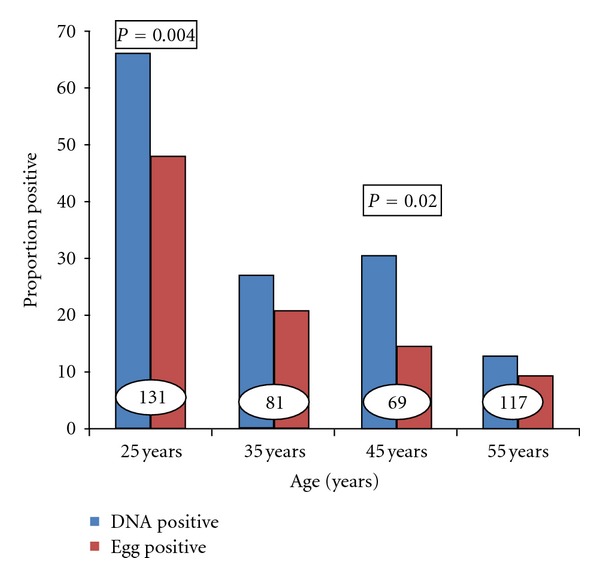
The proportion of *Schistosoma haematobium* cases diagnosed by demonstration of eggs or schistosome-specific DNA in the urine. 398 specimens were collected from villagers in western Nigeria where the parasite is endemic. Numbers in table = number examined,  *P*  values given where significant.

Analysis of the dataset described above (Figure 1) shows that the proportion of positive cases detected for each age group in the study was higher when *Dra*1 was detected by DNA amplification than when parasite eggs were observed for all age groups (*P* = 0.0005), although when stratified across age groups, significant differences were only seen in the 20–29- (*P* = 0.004) and 40–49- (*P* = 0.02) year-old age groups. The message from these studies is that if adult populations are examined for schistosome infection, if the only test applied is examination of urine for eggs, a significant number of people will be declared uninfected, yet they may yet be infected.

## 6. Conclusions and Future Directions

This example has shown that, for schistosomiasis, detection of parasite-specific DNA in urine is feasible, highly sensitive, and specific. To make this applicable in endemic countries where it may be difficult to operate thermocyclers and electrophoretic equipment, there is an alternative method that is already being promoted, namely, the loop-mediated isothermal amplification (LAMP) technique [16]. This method is well established as a viable and economical approach to DNA amplification and detection. Work needs to be done on refining the process for both species and developing it in a way that it can be put to use in the field as well in the hospital and diagnostic centre. The introduction of tests of high sensitivity and specificity will be valuable in monitoring the elimination programmes that are being carried out in many parts of the endemic world. In particular, when interventions are introduced, monitoring of treated individuals with very low parasitaemia will be necessary because of the risk of maintaining transmission to snails, and reinfection of the community. Detection of parasite-specific DNA will become an important means of identifying and hopefully eliminating risk foci.

There is no inference here that DNA detection is promoted to supersede other standard means of diagnosing schistosome infection. They are well tried and serve a role in most circumstances; however, as the need for more sensitive tests arise as outlined above, DNA detection adds another diagnostic which will expand the ability of the epidemiologist to collect data pertinent to any programme designed to eliminate the disease or warn clinicians of the potential problem of bladder cancer or some other sequel of this debilitating parasitic infection.
